# Veno-venous ECMO-assisted orthopedic stabilization for polytrauma with severe ARDS and refractory hypoxemia: a case report

**DOI:** 10.3389/fmed.2025.1688556

**Published:** 2026-01-23

**Authors:** Lin Liu, Kaiji Deng, Haifeng Tang, Yanjun Wang

**Affiliations:** Department of Emergency, Honghui Hospital, Xi’an Jiaotong University, Xi’an, Shaanxi, China

**Keywords:** ECMO, polytrauma, anticoagulation, ARDS, damage control orthopedics

## Abstract

This case report describes the successful integration of veno-venous extracorporeal membrane oxygenation (VV-ECMO) with physiologically optimized fracture fixation in a critically ill polytrauma patient who presented with life-threatening acute respiratory distress syndrome (ARDS). A 23-year-old male with bilateral femoral fractures, Gustilo IIIB open tibiofibular injury, left radius-ulna fractures, and refractory hypoxemia (PaO2/FiO2 40.5 mm Hg) underwent VV-ECMO initiation 1 h postinjury (total ECMO duration 144 h). Physiological optimization guided delayed surgical intervention on day 5, incorporating heparin-based anticoagulation and dynamic ECMO parameter modulation, resolving lactic acidosis (peak lactate 5.0 mmol/L on day 1 to 1.8 mmol/L preoperatively). The patient achieved successful decannulation by day 6 with satisfactory recovery at the 2-month follow-up [Short Musculoskeletal Function Assessment (SMFA) score 28.1, gait speed 1.2 m/s]. This case demonstrates the feasibility of a multidisciplinary protocol challenging traditional contraindications to surgery in severe ARDS patients and providing a replicable protocol for managing competing priorities of oxygenation and hemorrhage control.

## Introduction

The management of polytrauma patients with concurrent acute respiratory failure and complex orthopedic injuries presents a critical clinical challenge (1). Acute hypoxemic respiratory failure with refractory hypoxemia requires advanced management strategies, including VV-ECMO, which is distinct from veno-arterial ECMO (VA-ECMO) in its indications and applications (2). Recent evidence supports the feasibility of orthopedic procedures during ECMO support, highlighting the importance of integrated management for fracture stabilization (3, 4). The strategic timing of orthopedic surgery in these critically ill patients remains a key consideration, balancing the benefits of early stabilization against the risks of bleeding and systemic inflammation. This case addresses the gap in managing polytrauma with severe ARDS through a multidisciplinary approach.

## Case report

**FIGURE 1:**
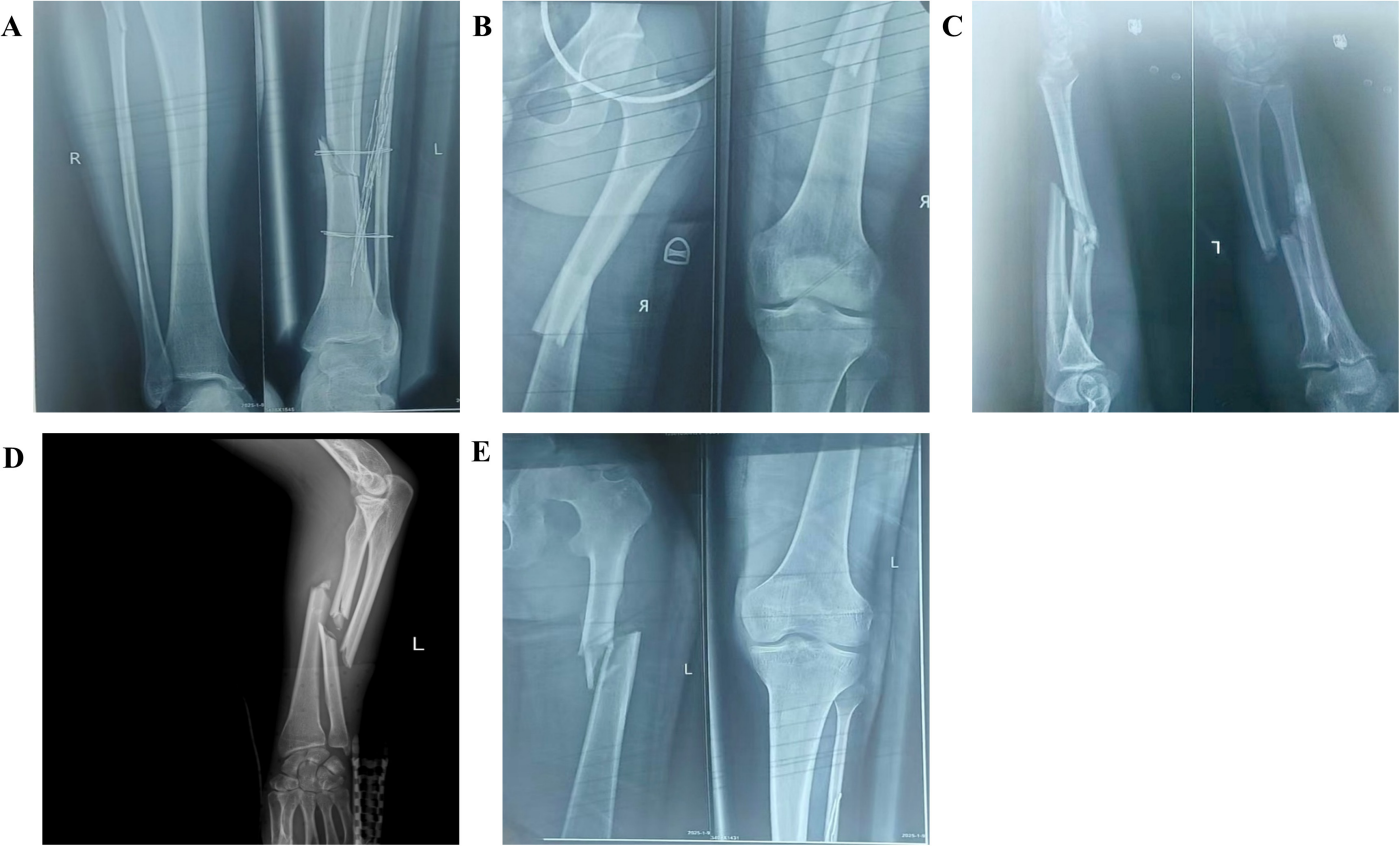
Preoperative radiographic findings: **(A)** Fracture of the right fibula (proximal, non-displaced); fracture of the left tibia (distal third, displaced); **(B)** Fracture of the right femur (midshaft, significantly displaced); **(C,D)** Left radial and ulnar fractures (midshaft, both displaced); **(E)** Fracture of the left femur (proximal third, displaced).

A 23-year-old male was transferred to our trauma center 9 h after a motor vehicle collision. Initial assessment revealed bilateral femoral shaft fractures, left Gustilo IIIB tibiofibular fractures, and ARDS with a PaO2/FiO2 ratio of 40.5 mm Hg (Figure 1). Chest imaging showed bilateral diffuse infiltrates consistent with ARDS Berlin criteria. Early surgical intervention was precluded by physiological barriers, including coagulopathy (INR 1.8, platelets 65 × 10^9^/L), lactic acidosis (pH 7.18, lactate 5.0 mmol/L), and hemodynamic instability requiring vasopressor support (5). Persistent hypoxemia despite a lung-protective ventilation strategy (tidal volume 220 mL (3.1 mL/kg predicted body weight), PEEP 14 cm H_2_O, plateau pressure 26 cm H_2_O, driving pressure 12 cm H_2_O) with transpulmonary pressure monitoring necessitated VV-ECMO cannulation via the right femoral vein and right internal jugular vein at 1 h postadmission. The selection of PEEP at 14 cm H2O was guided by an individualized lung-protective strategy.

**FIGURE 2:**
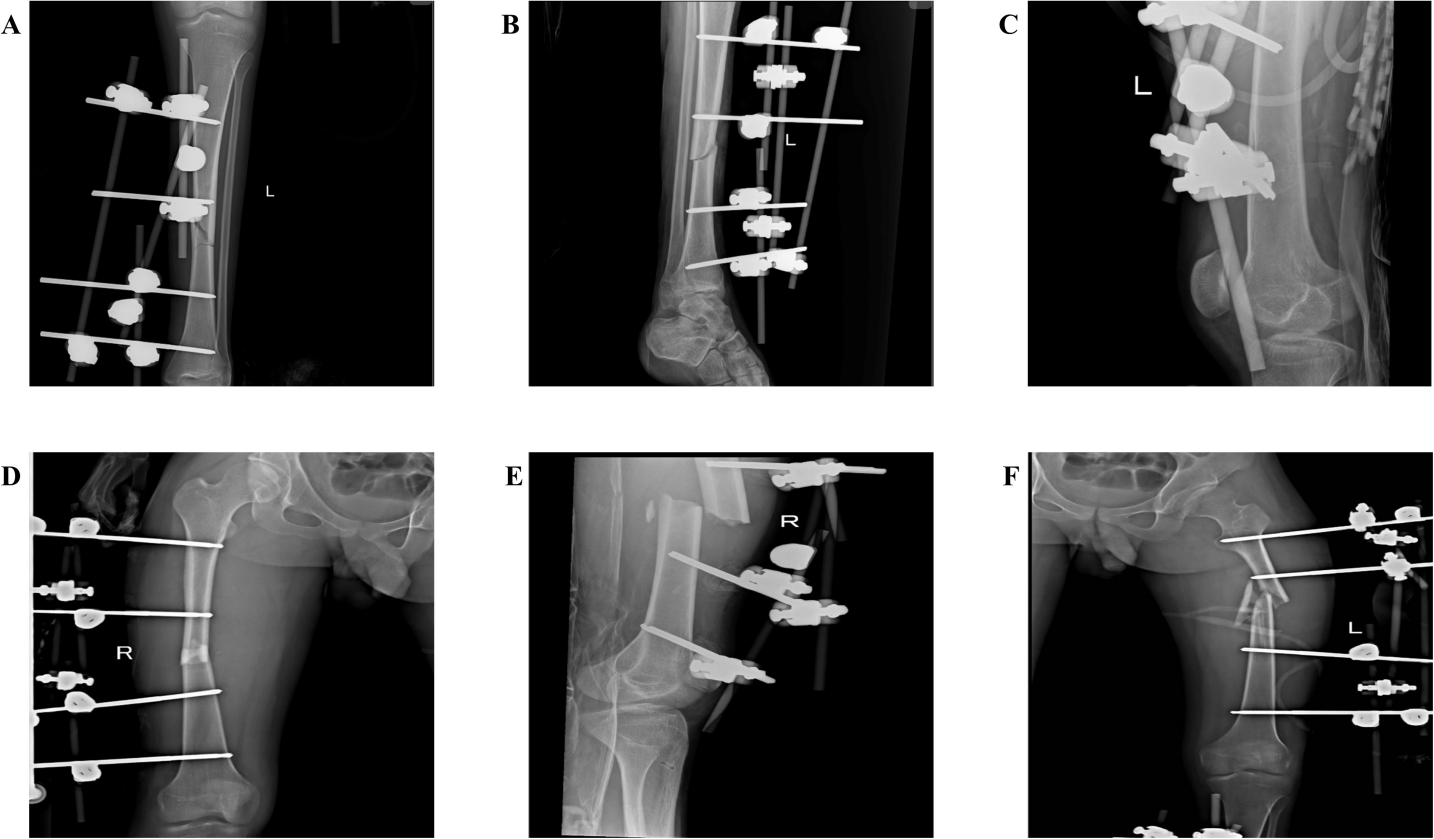
Postoperative external fixation for patients: **(A,B)** External fixation of the left tibia; **(C)** Left femoral external fixation; **(D,E)** Right femoral external fixation; **(F)** Left femoral external fixation.

**FIGURE 3:**
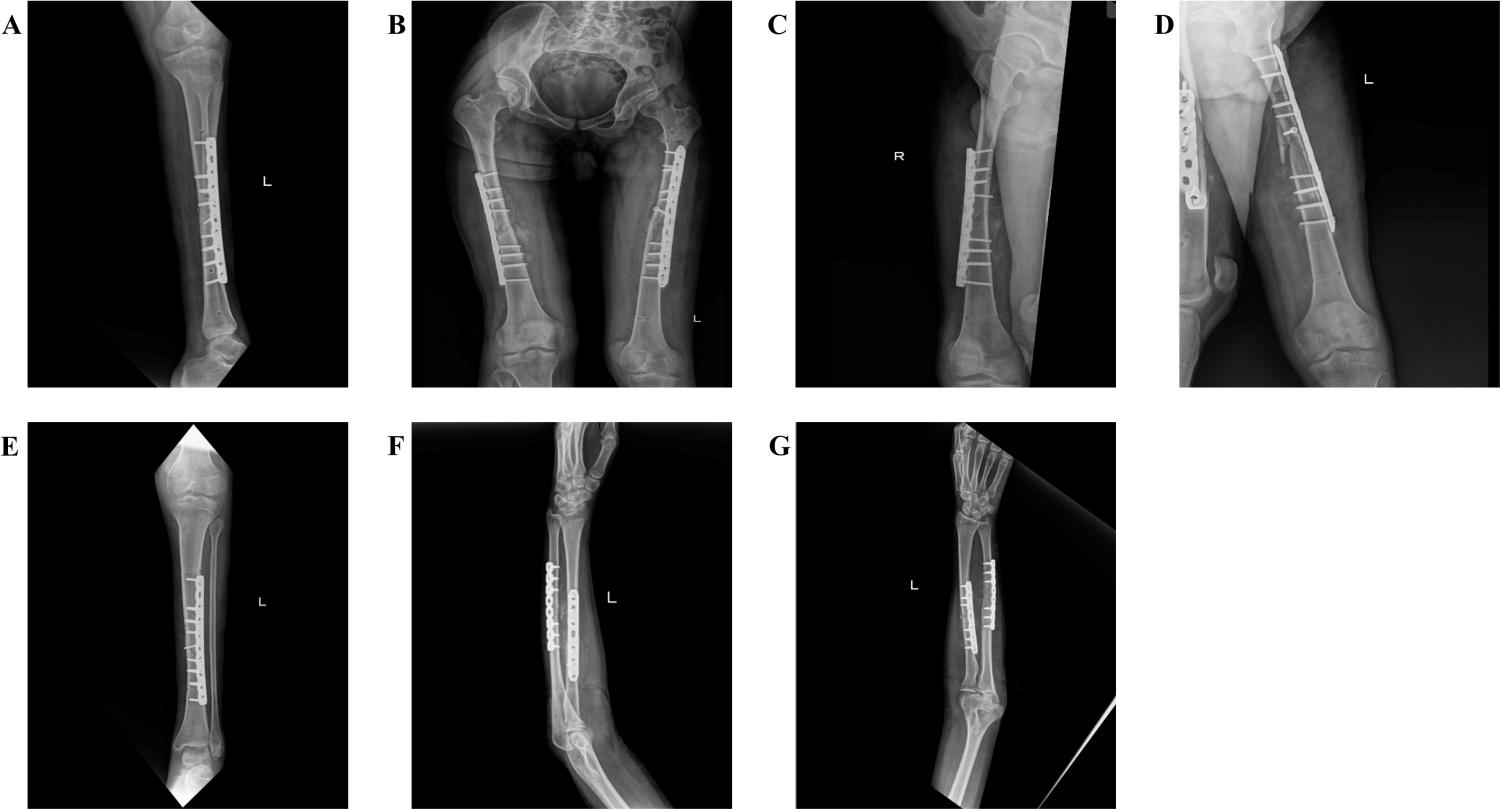
Postoperative internal fixation of patients: **(A)** Left tibial internal fixation; **(B)** Bilateral femoral internal fixation; **(C)** Right femoral internal fixation; **(D)** Left femoral internal fixation; **(E)** Left tibial internal fixation; **(F,G)** Left radial and ulnar internal fixation.

Anticoagulation was initiated with unfractionated heparin infusion (18 U/kg/h), adjusted to maintain ACT for 180–200 s and anti-Xa at 0.3–0.7 IU/mL, with fibrinogen supplementation maintained at 2.8 g/L. Platelet count recovery (> 100 × 10^9^/L) and fibrinogen levels (> 2.5 g/L) were confirmed prior to surgery. Physiological optimization by postinjury day 5 (pH 7.32, platelet count 112 × 10^9^/L, fibrinogen 2.8 g/L) permitted closed reduction and external fixation of the bilateral femurs and left tibia. Intraoperative ECMO management included dynamic sweep gas adjustment from 4.3 L/min during dissection to 4.5 L/min during reduction, maintaining SvO2 > 75% throughout the 30-min procedure with minimal blood loss (50 mL) attributed to percutaneous fixation techniques (Figure 2). The patient was switched to prone position ventilation after surgery to promote sputum discharge (6). Successful decannulation occurred on day 6 following pulmonary recovery [PaO2/FiO2 320 mm Hg on pressure support ventilation (PSV 10 cm H_2_O, PEEP 8 cm H_2_O)]. Staged internal fixation was successfully completed by postoperative day 30 (Figure 3). At the 2-month follow-up evaluation, the patient demonstrated significant functional recovery, with independent ambulation at a gait speed of 1.2 m/s, Timed Up and Go test of 11 s, and hip flexion range of motion 0–115°. Additionally, the short Musculoskeletal Function Assessment (SMFA) score improved to 28.1, and C-reactive protein (CRP) normalized from 68 to 12 mg/L. The patient achieved independent ambulation without clinical signs of deep vein thrombosis (DVT).

## Discussion

This intervention redefines two critical paradigms in contemporary trauma care. First, it establishes the viability of orthopedic procedures under VV-ECMO support for patients with life-threatening ARDS (PaO2/FiO2 < 50 mm Hg), a population traditionally excluded from surgical candidacy. Our intraoperative ECMO protocol, featuring real-time sweep gas titration (4.3→4.5 L/min) to sustain cerebral oxygenation (SvO2 > 75%) during fracture manipulation, synthesizes cardiovascular (7) and transplant surgery (8) techniques into a novel trauma application. Second, this case resolves the historical dilemma between anticoagulation and surgical hemostasis through thrombin-targeted therapy.

In contrast to previous reports advocating heparin-free strategies (9, 10), we observed that controlled heparinization (18 U/kg/h) coupled with fibrinogen supplementation (> 2.5 g/L) achieved hemostasis without circuit thrombosis. Despite emerging evidence favoring direct thrombin inhibitors for thromboprophylaxis (11–14), our heparin-based regimen (18 U/kg/h, ACT 180–200 s) with fibrinogen supplementation (> 2.5 g/L) achieved dual success: negligible intraoperative bleeding (50 mL) and circuit patency, aligning with ELSO guidelines for trauma-ECMO anticoagulation (15). This cost-efficacy advantage persists even when factoring in potential platelet transfusion requirements, as demonstrated in recent pharmacoeconomic analyses (12).

Recent evidence from a randomized controlled pilot study by Pintado et al. (16) demonstrated that, compared with conventional FiO2-guided approaches, compliance-guided PEEP in ARDS patients significantly increased the number of organ dysfunction-free days. This physiological optimization protocol aligns with our dynamic ECMO management, where maintaining a plateau pressure below 30 cm H2O while achieving optimal compliance proved critical for mitigating ventilator-induced lung injury in this polytrauma-ARDS scenario. The strategic delay to postinjury day 5 challenges conventional DCO (17), guided by a triad of physiological parameters: acid–base equilibrium restoration (pH 7.32), hemostatic recovery (platelets > 100 × 10^9^/L), and pulmonary compliance improvement (ΔP 18 cm H2O). This “resuscitation window” concept gains biological plausibility from preclinical models showing that delayed fixation reduces secondary inflammatory cascades in thoracic trauma (18). The dual osteogenic mechanism of ECMO—sustaining tissue oxygenation (pO2 > 60 mm Hg) to prevent hypoxia-induced osteoblast dysfunction (19) and curtailing ventilator-induced IL-6 elevation (6 vs. 14 days) that impairs BMP-2 signaling (20)—likely contributed to accelerated functional recovery, as evidenced by independent ambulation (gait speed 1.2 m/s) and improved SMFA scores (28.1), indicating mild dysfunction.

Neurological protection constituted a cornerstone of our protocol, integrating multimodal monitoring (cerebral oximetry, MAP regulation) to mitigate ECMO-associated encephalopathy risks (21). The absence of neurological sequelae underscores the feasibility of real-time neuromonitoring in trauma-ECMO, although standardized protocols remain elusive. While single-center experience and survivor bias limit generalizability, this case provides a framework for multicenter validation using advanced biomarkers (thromboelastography) and cytokine profiling to optimize immunomodulatory strategies.

## Conclusion

This case demonstrates that VV-ECMO can enable safe orthopedic stabilization in polytrauma patients with severe ARDS.

## Data Availability

The original contributions presented in this study are included in this article/supplementary material, further inquiries can be directed to the corresponding authors.

## References

[1] Pape HC Tornetta P Tarkin I Tzioupis C Sabeson V Olson SA. Timing of fracture fixation in multitrauma patients: the role of early total care and damage control surgery. *J Am Acad Orthop Surg*. (2009) 17:541–9. 10.5435/00124635-200909000-00001 19726738

[2] Combes A Hajage D Capellier G Demoule A Lavoué S Guervilly C Extracorporeal membrane oxygenation for severe acute respiratory distress syndrome. *N Engl J Med*. (2018) 378:1965–75. 10.1056/NEJMoa1800385 29791822

[3] Kakalecik J Frantz AM Talerico MT Krupko TA Hagen JE Patrick MR. Orthopaedic fracture surgery in polytraumatized patients while on extracorporeal membrane oxygenation (ECMO): a report of two cases. *Trauma Case Rep*. (2024) 51:101020. 10.1016/j.tcr.2024.101020 38633378 PMC11021949

[4] McCormick WF Yeager MT Morris C Johnston TR Schick S He JK The effect of extracorporeal membrane oxygenation in patients with multiple orthopaedic injuries. *J Am Acad Orthop Surg*. (2024) 32:904–9. 10.5435/JAAOS-D-24-00026 38833727

[5] Scalea TM Boswell SA Scott JD Mitchell KA Kramer ME Pollak AN. External fixation as a bridge to intramedullary nailing for patients with multiple injuries and with femur fractures: damage control orthopedics. *J Trauma.* (2000) 48:613–21; discussion 621–3. 10.1097/00005373-200004000-00006 10780592

[6] Guérin C Albert RK Beitler J Gattinoni L Jaber S Marini JJ Prone position in ARDS patients: Why, when, how and for whom. *Intensive Care Med*. (2020) 46:2385–96. 10.1007/s00134-020-06306-w 33169218 PMC7652705

[7] Brewer JM Tran A Yu J Ali MI Poulos CM Gates J ECMO after cardiac surgery: a single center study on survival and optimizing outcomes. *J Cardiothorac Surg*. (2021) 16:264. 10.1186/s13019-021-01638-0 34538270 PMC8451085

[8] Zhang C Wang Q Lu A. ECMO for bridging lung transplantation. *Eur J Med Res*. (2024) 29:628. 10.1186/s40001-024-02239-y 39726046 PMC11670462

[9] Arlt M Philipp A Voelkel S Rupprecht L Mueller T Hilker M Extracorporeal membrane oxygenation in severe trauma patients with bleeding shock. *Resuscitation*. (2010) 81:804–9. 10.1016/j.resuscitation.2010.02.020 20378236

[10] Muellenbach RM Kredel M Kunze E Kranke P Kuestermann J Brack A Prolonged heparin-free extracorporeal membrane oxygenation in multiple injured acute respiratory distress syndrome patients with traumatic brain injury. *J Trauma Acute Care Surg*. (2012) 72:1444–7. 10.1097/TA.0b013e31824d68e3 22673280

[11] Kaseer H Soto-Arenall M Sanghavi D Moss J Ratzlaff R Pham S Heparin vs bivalirudin anticoagulation for extracorporeal membrane oxygenation. *J Card Surg*. (2020) 35:779–86. 10.1111/jocs.14458 32048330

[12] Fisser C Winkler M Malfertheiner MV Philipp A Foltan M Lunz D Argatroban versus heparin in patients without heparin-induced thrombocytopenia during venovenous extracorporeal membrane oxygenation: a propensity-score matched study. *Crit Care*. (2021) 25:160. 10.1186/s13054-021-03581-x 33910609 PMC8081564

[13] Li MJ Shi JY Zhang JH. Bivalirudin vs. heparin in paediatric and adult patients on extracorporeal membrane oxygenation: a meta-analysis. *Br J Clin Pharmacol*. (2022) 88:2605–16. 10.1111/bcp.15251 35098565

[14] Liu L Liu F Tan J Zhao L. Bivalirudin versus heparin in adult and pediatric patients with extracorporeal membrane oxygenation therapy: a systematic review and meta-analysis. *Pharmacol Res*. (2022) 177:106089. 10.1016/j.phrs.2022.106089 35065202

[15] McMichael ABV Ryerson LM Ratano D Fan E Faraoni D Annich GM. 2021 ELSO adult and pediatric anticoagulation guidelines. *ASAIO J*. (2022) 68:303–10. 10.1097/MAT.0000000000001652 35080509

[16] Pintado MC de Pablo R Trascasa M Milicua JM Rogero S Daguerre M Individualized PEEP setting in subjects with ARDS: a randomized controlled pilot study. *Respir Care*. (2013) 58:1416–23. 10.4187/respcare.02068 23362167

[17] Pape HC Rixen D Morley J Husebye EE Mueller M Dumont C Impact of the method of initial stabilization for femoral shaft fractures in patients with multiple injuries at risk for complications (borderline patients). *Ann Surg.* (2007) 246:491–9; discussion 499–501. 10.1097/SLA.0b013e3181485750 17717453 PMC1959352

[18] Blair JA Kusnezov N Fisher T Prabhakar G Bader JO Belmont PJ. Early stabilization of femur fractures in the setting of polytrauma is associated with decreased risk of pulmonary complications and mortality. *J Surg Orthop Adv.* (2019) 28:137–43. 10.3113/JSOA.2019.0137 31411960

[19] Yin Q Yang H Fang L Wu Q Gao S Wu Y Fibroblast growth factor 23 regulates hypoxia-induced osteoblast apoptosis through the autophagy-signaling pathway. *Mol Med Rep*. (2023) 28:199. 10.3892/mmr.2023.13086 37711045 PMC10540001

[20] Huang RL Sun Y Ho CK Liu K Tang QQ Xie Y IL-6 potentiates BMP-2-induced osteogenesis and adipogenesis via two different BMPR1A-mediated pathways. *Cell Death Dis*. (2018) 9:144. 10.1038/s41419-017-0126-0 29396550 PMC5833364

[21] Khanduja S Kim J Kang JK Feng CY Vogelsong MA Geocadin RG Hypoxic-ischemic brain injury in ECMO: pathophysiology, neuromonitoring, and therapeutic opportunities. *Cells*. (2023) 12:1546. 10.3390/cells12111546 37296666 PMC10252448

